# Acupuncture for pain control after degenerative lumbar spine surgery

**DOI:** 10.1186/s40001-022-00797-7

**Published:** 2022-09-01

**Authors:** Bo-An Chen, Wen-Chun Deng, Mao-Yu Chen

**Affiliations:** 1 Department of Neurosurgery, Chang Gung Memorial Hospital, Linkou, Taiwan; 2 Department of Neurosurgery, Chang Gung Memorial Hospital, Keelung, Taiwan

**Keywords:** Acupuncture, Degenerative spine disease, Non-steroidal anti-inflammatory drugs, Acetaminophen, Opioid

## Abstract

**Background:**

Wound pain after surgery for lumbar spine disease may interfere with patients’ recovery. Acupuncture is commonly used for pain management, but its efficacy for postoperative pain control is unclear. This study aimed to evaluate the effectiveness of acupuncture for adjuvant pain control after surgery for degenerative lumbar spine disease.

**Methods:**

We retrospectively reviewed the records of consecutive patients who received surgery for degenerative lumbar spine disease at our institution from 2013 to 2014. Surgical procedures included open laminectomy, discectomy, and trans-pedicle screw instrumentation with posterior-lateral fusion. Patients were grouped by pain control methods, including routine analgesia, patient-controlled analgesia (PCA), and acupuncture. The routine analgesia group received oral acetaminophen/non-steroidal anti-inflammatory drugs with meperidine as needed for immediate pain control. The PCA group received a basal dose of morphine and subsequent user-demand doses. The acupuncture group received acupuncture every other day after surgery.

**Results:**

Ninety-six patients were included, of whom 37 received acupuncture, 27 received PCA, and 32 received routine analgesics for pain control. Visual analog scale (VAS) pain scores in all 3 groups decreased significantly, and to the same degree, from the first postoperative day to the second day. No significant differences were found in VAS scores over the next 6 postoperative days; however, the scores of patients treated with PCA were slightly but still significantly higher (*p* = 0.026) on postoperative day 4 than scores of patients treated with acupuncture and traditional analgesia, a difference likely due to PCA being discontinued on postoperative day 3. No major complications were noted in the acupuncture group, but 2 patients dropped out because of fear of needle insertion.

**Conclusions:**

Acupuncture may be as effective as traditional analgesia and PCA for adjuvant pain control after surgery for degenerative lumbar spine disease.

## Background

Low back pain caused by degenerative spine disease is a common health issue for which individuals seek medical care. Ravindra et al. [1] reported that about 266 million persons experience low back pain each year worldwide. Degenerative spine disease is treated with non-surgical and surgical approaches. The non-surgical options include physical therapy, pharmacotherapy, and epidural injections [2]. If non-surgical methods fail, surgery may be required, although the indications for surgery for degenerative spine disease are not established.

Acetaminophen and non-steroid anti-inflammatory drugs (NSAIDs) are the most frequently used first-line oral analgesics for postoperative pain control. However, side effects, such as liver toxicity with acetaminophen and peptic ulcer disease or cardiovascular events with NSAIDs, are of concern [3]. Opiate drugs are widely used for control of more severe pain, but they also have side effects, such as nausea or vomiting. Opiates in combination with acetaminophen or NSAIDs are sometimes used to achieve control of pain with minimal side effects [3]. Continuous infusion of meperidine or morphine through patient-controlled analgesia (PCA) is also widely used for management of pain.

Acupuncture is another approach for pain control. In traditional Chinese medicine (TCM), acupuncture helps energy, or “qi,” flow through the body smoothly and helps keep yin and yang balanced [4]. Over the years, many reports have reviewed the theories and indications of acupuncture for pain control. In recent years, systematic reviews, meta-analyses, and randomized trials have been conducted for control of postoperative pain using acupuncture. In a systematic review and meta-analysis, Wu et al. [5] evaluated acupuncture, electroacupuncture, and acupoint electrical stimulation for managing acute postoperative pain. Overall, they found that these techniques improved postoperative pain on the first day after surgery and reduced opioid use, and concluded that acupuncture is an appropriate adjuvant therapy in treating postoperative pain. Madsen et al. [6] conducted a systematic review of randomized trials of acupuncture treatment for pain, including postoperative pain. Lam et al. [7] described a protocol for a randomized trial of combined electroacupuncture and auricular acupuncture for postoperative pain after abdominal surgery for gynecological diseases. Despite these reports, the literature is sparse on evaluation of acupuncture for the management of postoperative wound pain.

The surgical approach for spinal disease has evolved significantly in recent years. In our hospital during 2013–2014, our surgical procedure for degenerative lumbar spine disease was standardized into open laminectomy with or without pedicle screws fixation. As a result, potential biases caused by different surgical approaches were able to be minimized. On the other hand, TCM at our hospital began to promote acupuncture for pain control and cooperated with various other departments in our hospital during that period. In this study we evaluated the efficacy of acupuncture as adjuvant therapy for the control of pain after surgery for degenerative lumbar spine disease during this time period.

## Materials and methods

### Patients and study design

In this retrospective study, all medical records data of patients who received traditional open surgery (minimally invasive procedures or vertebroplasty not included) for degenerative spine disease at our institution from 2013 to 2014 were analyzed. The study protocol was approved by the Institutional Review Board (IRB) of our Foundation (IRB reference number: 202000811B0). During the study, 120 lumbar spine surgeries were performed, and twelve patients with trauma, tumors, or infection were excluded. An additional 12 patients were also excluded for discharge against medical advice (*n* = 1), incomplete medical records (*n* = 6), equivocal trauma history (*n* = 2), or incomplete acupuncture course (*n* = 3). Surgical procedures included standard open laminectomy, discectomy, and posterior-lateral fusion with trans-pedicle screw instrumentation. All surgical procedures were standardized under the hospital system.

Finally, the data of 96 consecutive patients were included as the analytic sample. All patients received regular acetaminophen 500 mg/ibuprofen 400 mg four times a day postoperatively, categorized according to their adjuvant pain control method. (1) The routine analgesia (NA) group received oral acetaminophen/NSAIDs with meperidine (25 mg intramuscular injection) every 4 h as needed for immediate pain control (*n* = 32). (2) The PCA group (*n* = 27) received a basal dose of morphine (1 mg/h) plus user-demand dosing beginning immediately postoperatively in the recovery room until the morning of postoperative day 3. 3) The acupuncture (AC) group (*n* = 37), received acupuncture given by our Chinese medical physician every other day after the surgery. The choice for pain control was not randomly assigned. Instead, after thoroughly explaining to the patients about the advantages and disadvantages of different pain control methods, the method was determined by the patients. This was done mainly because PCA and acupuncture were not covered by the Taiwan national health insurance (NHI). Although this study was not a multiple treatment modalities trial, patients could still have additional medications for pain control if they experienced intolerable pain. Fourteen of 32 patients in the NA group and 7 of 37 patients in the AC group received additional analgesia. Acupuncture was administered at 7 acupoints: GB-34 (Yanglingquan), Ki-3 (Taixi), Du-20 (Baihui), Ex-UE7 (Yaotongxue), Bl-60 (Kunlun), St-36 (Zusanli), and SI-3 (Houxi). The acupuncture group was further divided into 2 subgroups for additional analysis: subgroup A (*n* = 23) received acupuncture prior to surgery on the day of the operation, and then 24 h after surgery and every other day thereafter; subgroup B (*n* = 14) received acupuncture every other day beginning 24 h after surgery. Twenty-eight patients discontinued acupuncture two days before discharge and nine patients received acupuncture one day before discharge. No patients received acupuncture on the day of discharge.

The primary outcome was pain as recorded using the visual analog scale (VAS) with these cut points: 0, no pain; 1–4, mild pain; 5–7, moderate pain; 8–10, severe pain, worst imaginable pain [8]. VAS scores were recorded 4 h postoperatively, and 6:30 am each day beginning on postoperative day 1. Resolution of the chief complaint (e.g., back pain) was recorded as the surgical outcome according to the categories of complete remission, much improved, modestly improved, unchanged, and worse. Complications assessed were bleeding, infection, dizziness, or any other treatment-related adverse effects.

### Statistical analysis

All statistical analyses were carried out using IBM SPSS statistical software version 24 for Windows (IBM Corp., Armonk, New York, USA). Comparisons of pre- and postoperative parametric data of each group were performed using the D’Agostino and Pearson test for normality. Comparison of parametric results between the 3 groups was performed using ANOVA. The non-parametric (Mann–Whitney test) was used for the subgroup who received preoperative acupuncture (Pre AC) and those who did not (No Pre AC). Contingency results, including improvement of the chief complaint, sex, and surgical methods, were compared using Chi-square analysis. All statistical assessments were 2 tailed, and a value of *p* < 0.05 was considered to indicate a statistically significant difference.

## Results

### Patients’ demographic and clinical characteristics

Data of 108 patients were initially retrieved and 97 patients who met the inclusion criteria were identified. Recruited patients were categorized into different pain control groups based on their own preferences. No significant differences were found between the 3 groups in sex, age, spinal segment operated on, surgical method, blood loss, operation time, and preoperative VAS pain score (Table 1).

**Table 1:** Patients’ characteristics and surgical procedures

	Total (*N* = 96)	AC (*n* = 37)	PCA (*n* = 27)	NA (*n* = 32)	*p*
Sex ratio					0.724
M/F	60	68	59	45	
Age	62 (17)	62 (14)	61 (15)	61.5 (20.75)	0.782
Blood loss (mL)	400 (450)	450 (512.5)	250 (500)	350 (400)	0.446
Operation time (min)	160 (70)	155 (65)	160 (70)	160 (82.5)	0.393
Preoperative VAS pain score	6 (1)	6.5 (2.25)	6 (1)	6 (2)	0.481
Segments operated	4 (1)	4 (1)	3 (1)	4 (1)	0.638
Surgical method (%)					
Laminectomy	17.6	21.2	7.4	21.9	
Laminectomy + TPS	55.1	60.2	55.6	50	
Laminectomy + + Discectomy	20.7	13.3	25.9	25	
Laminectomy + + TPS and discectomy	6.3	5.3	11.1	3.1	

Data are reported as numbers (percentage) or medians (IQR)

*AC* acupuncture, *PCA* patient-controlled analgesia, *NA* routine NSAIDs analgesics, *TPS* trans-pedicle screw, *VAS* visual analog scale

*p < 0.05

### Postoperative pain control assessment

**Fig. 1:**
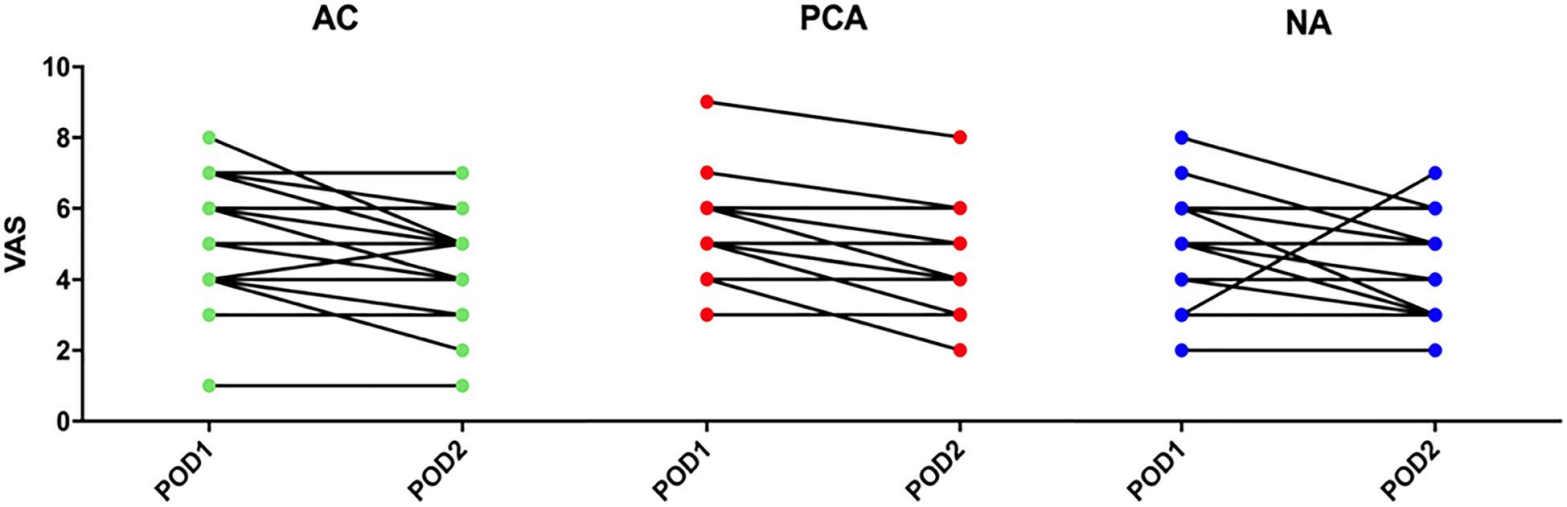
Comparison of VAS pain scores between postoperative day 1 and postoperative day 2. Paired *t* test was used for analysis. Significant decreases in VAS scores were shown in all 3 groups from the first to the second postoperative day (all, *p* < 0.05)

As shown in Fig. 1, the VAS pain scores in all three groups decreased significantly from the first postoperative day to the second day, and no significant differences were found in the amount of decrease.

**Fig. 2:**
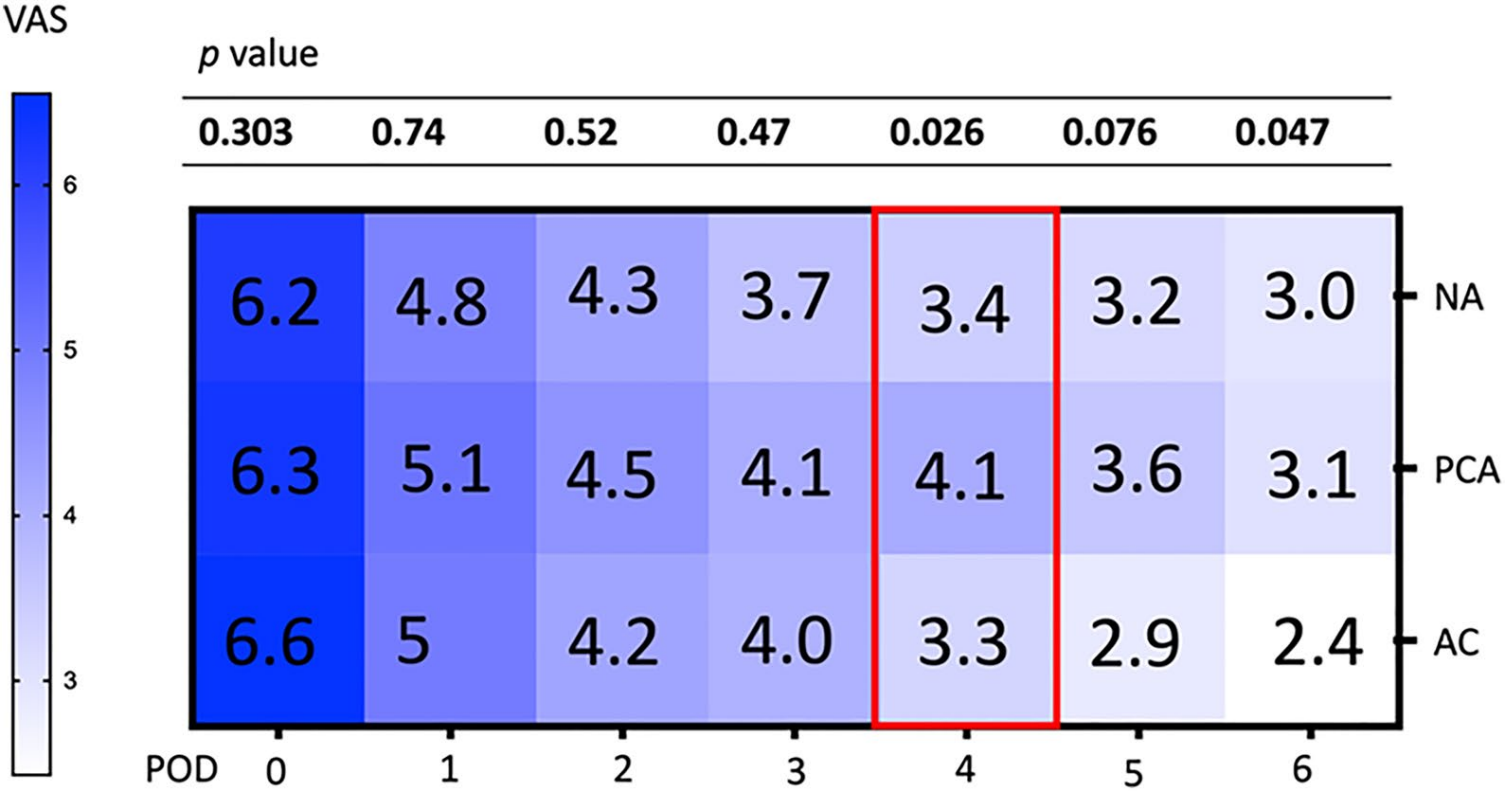
Daily changes of VAS pain scores in the first 6 days after surgery (Data presented as median and statistical analysis by ANOVA.) At postoperative day 4, the VAS scores were significantly higher in the PCA group than in the NA and AC groups (*p* = 0.026). By day 6, the VAS scores in the AC group were significantly lower than in the other 2 groups (*p* = 0.047)

The daily changes in VAS pain scores over the first postoperative week are illustrated in Fig. 2. The decline in VAS pain scores was similar among the three groups, with only minor differences. On day 4, the VAS score was significantly higher in the PCA group than in the other two groups (*p* = 0.026), and it was borderline significantly different on day 5 (*p* = 0.076). By day 6, the VAS score in the PCA group had declined to that of the NA group, but VAS was significantly lower than those in both the PCA group and the NA group (*p* = 0.0471). Due to persistent pain, 4 patients requested acupuncture after discontinuation of PCA on postoperative day 3; 3 patients received acupuncture on postoperative day 4, and 1 patient received acupuncture on postoperative day 5.

**Fig. 3:**
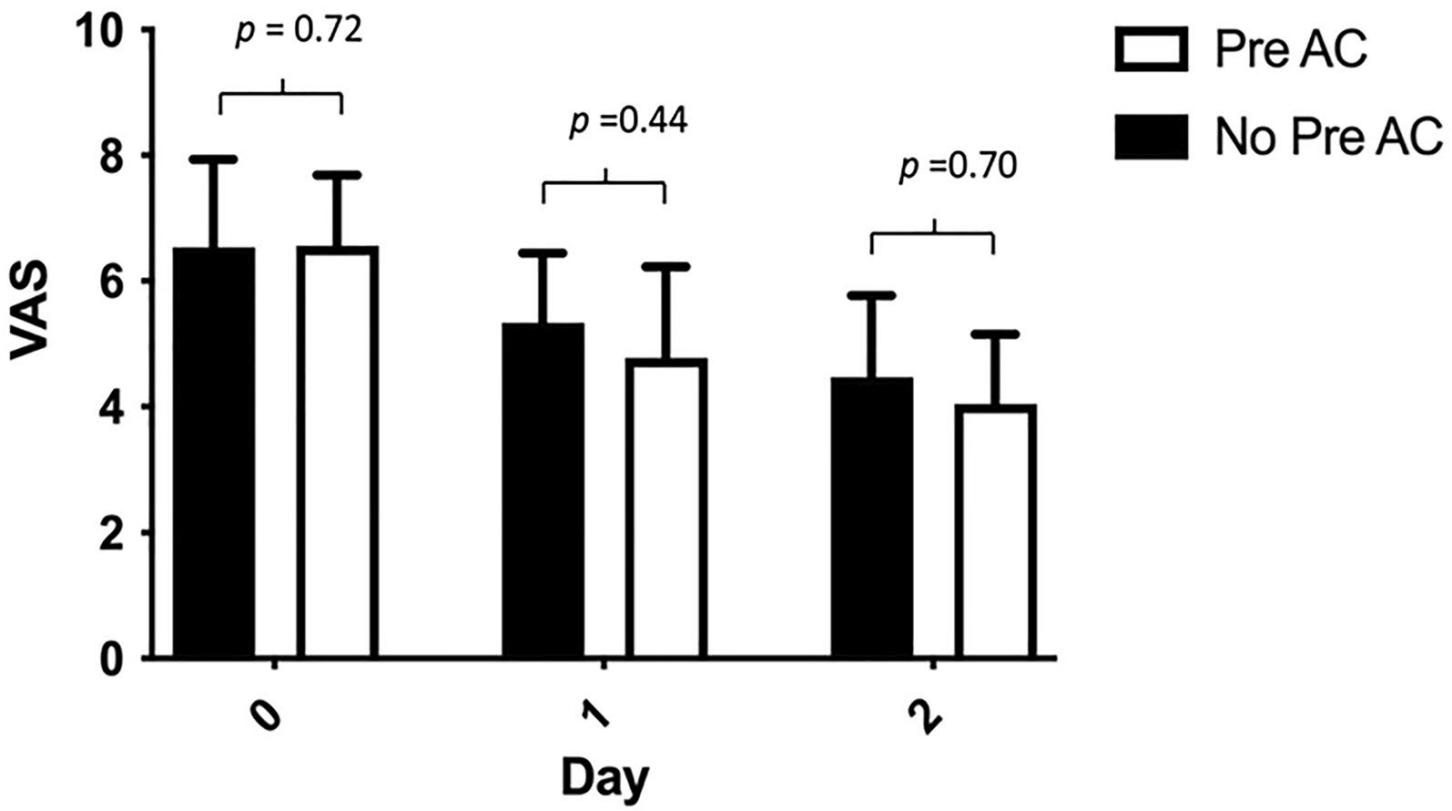
VAS pain scores on postoperative days 1 and 2 for patients who received preoperative acupuncture (Pre AC) and those who did not (No Pre AC). No statistically significant differences were noted between the groups (day 1 *p* = 0.44, day 2 *p* = 0.70)

Subgroup analysis of the AC group indicated that VAS scores on postoperative days 1 and 2 were not significantly different in patients who received preoperative acupuncture and those who did not (postoperative day 1 *p* = 0.438, postoperative day 2 = 0.695) (Fig. 3). The VAS in both subgroups were significantly lower between pre- and postoperative days 1 and 2.

### Surgical outcomes

Surgical outcomes were assessed on the day of discharge and are summarized in Table 2. Twenty-six patients (68.4%) in the AC group reported complete resolution or much improvement of their pain after spine surgery, as compared with 51.9% in the PCA group and 37.5% in the routine analgesic group (*p* = 0.024). When compared individually by using Chi-square test with Fischer exact test, the *p* value for outcomes was 0.192 between the AC and PCA groups and 0.0082 between the AC and NA groups. These results showed better outcomes in the AC group compared to those in the routine analgesia group. Outcome of “modestly improved” and “worse” were not significantly different between the three groups. Only one patient in the AC group received a single dose of meperidine (25 mg intramuscular) during the treatment course due to intolerable pain before the start of acupuncture. Four patients received AC after stopping PCA because of persistent pain. The characteristics of the three analgesia methods are compared in Table 3.

**Table 2:** Surgical outcomes

	AC (*n* = 37)	PCA (*n* = 27)	NA (*n* = 32)	*p*
	26 (68.42)	14 (51.85)	12 (37.50)	0.024*
Complete remission	3 (8.11)	0 (0)	1 (3.13)	
Much improved	23 (62.16)	14 (51.85)	11 (34.38)	
	11 (29.73)	13 (48.15)	20 (62.50)	
Modestly improved	10 (27.02)	11 (40.74)	20 (62.50)	
Unchanged	1 (2.70)	2 (7.41)	0 (0)	
Worse	0 (0)	0 (0)	0 (0)	

Data reported as numbers (percentages)

*AC* acupuncture, *PCA* patient-controlled analgesia, *NA* routine NSAIDs analgesics

*p < 0.05

**Table 3:** Characteristics of 3 postoperative analgesia methods

	AC	PCA	NA
Effectiveness in relieving pain	Good	Good	Moderate
Duration of relief	Short	Prolonged	Short
Cost	Low	High	Low
Safety	High	Questionable	Questionable
Other	Fear of needle	Rebound pain	

*AC* acupuncture, *PCA* patient-controlled analgesia, *NA* routine NSAIDs analgesics

### Complications

No major complications (bleeding, infection, or dizziness) occurred in the AC group. However, 2 patients requested another form of analgesia because of fear of needle placement.

## Discussion

The present study compared acupuncture, routine analgesic medications (oral acetaminophen/NSAIDs with meperidine), and PCA for adjuvant control of postoperative pain after open surgery for degenerative spine disease. The most important finding was that acupuncture was as effective as the other two methods of pain control. The decline in VAS pain scores between postoperative days 1 and 2 was not significantly different between the three groups. Over the next six postoperative days, pain scores were essentially similar among the three groups, except that pain scores in the PCA group on postoperative day 4 were slightly higher than those of the other two groups. Interestingly, significantly more patients treated with acupuncture reported complete resolution or much improvement of their back pain after surgery than did patients in the other two groups.

Variables in the pain control regimens and the surgeries of the three groups in this study complicate the comparison of the results of the different pain control methods (e.g., use of NSAIDs of unknown amounts, for all patients and unknown subgroup differences in analgesia methods used for various types of surgery). Nonetheless, acupuncture achieved greater pain control than the other two methods, without the disadvantages of side effects of opioids or NSAIDs or the cost of PCA. Acupuncture also was found to be safe, with no recognized adverse effects. Acupuncture has a disadvantage, though, in that a small number of patients are afraid of needle placement. Until rigorous controlled, prospective studies unequivocally establish acupuncture as safe and effective in the management of postoperative pain after surgery for degenerative spine disease, it will remain an attractive option but without proven superiority over conventional methods.

Achieving great improvement in postoperative pain relief in patients undergoing spinal surgery is often a challenge. Therefore, complementary approaches, including chiropractic therapy, physiotherapy, massage, exercise, herbal medicine, and acupuncture, in addition to conventional analgesic are usually integrated into the rehabilitation programs. However, the effectiveness of these complementary therapies in improving postoperative pain control is not well defined.

Acupuncture has been gaining popularity for the treatment of pain arising from various causes, particularly including low back pain. Xiang et al. [9] conducted a systematic review and meta-analysis, reporting that acupuncture was associated with greater immediate pain relief than was sham acupuncture or analgesic injections. The authors, however, stated that more studies with rigorous design and methodologies were needed to confirm the findings. On the other hand, acupuncture may have the benefit of reducing opioid addiction. In a study aimed to investigate the effects of preoperative electroacupuncture on postoperative pain and opioid-related side effects, Lin et al. [10] showed that both low and high electroacupuncture can reduce postoperative analgesic requirements and associated side effects in patients undergoing lower abdominal surgery. A randomized controlled study found that auricular acupuncture reduced the amount of opioids used during PCA after total hip arthroplasty [11]. In addition, in a meta-analysis including 39 randomized clinical trials with 2391 patients, the authors indicated that acupuncture significantly delayed opioid use after total knee arthroplasty, with 20.84 to 71.50 min to the first PCA [12]. Additionally, a systematic review and meta-analysis found that acupuncture plus drug therapy was more effective than conventional drug therapy alone for relief of cancer-related pain; however, the authors considered that the results were compromised by poor quality of data [13]. Nevertheless, increasing evidence has shown that acupuncture can effectively decrease opioid dependence and avoid addiction to opioids [14]. The present study expanded these findings and further showed that acupuncture was as effective as PCA for patients who received morphine at a basal dose plus user-demand doses in the control of postoperative pain after degenerative lumbar spine surgery. Taken together, acupuncture may replace opioids during the postoperative recovery period, and may help achieve the ultimate goal of reducing the opioid crisis.

Acupuncture also has economic benefits. The total cost of one PCA treatment course is approximately 300 US dollars, while the total cost of acupuncture is only about 30 US dollars. Such cost-effectiveness of acupuncture is also in accordance with other studies. In a systematic review that included 7 cost-utility analyses and 1 cost-effectiveness analysis concerning acupuncture for chronic pain, the authors showed that the cost per quality-adjusted life-year gained was below the commonly quoted thresholds used by the UK National Institute for Health and Clinical Excellence for “willingness to pay” [15]. In another cost-effectiveness analysis regarding non-pharmacological treatments for osteoarthritis of the knee, acupuncture was suggested to be the most cost-effective option when analysis was limited to high-quality studies [16]. In addition, in a report published in 2018, Lim et al. suggested that acupuncture is one of the most used non-pharmacologic pain-relieving techniques for low back pain due to its low adverse effects and high cost-effectiveness [17]. Furthermore, a study from the Center for Health Information and Analysis found that acupuncture can save $35 480, $32 000, $9 000, and $4 246 per patient for migraine, angina pectoris, severe osteoarthritis, and carpal tunnel syndrome, respectively [18]. Thus, it appears that by using acupuncture, patients can save money and successfully manage their pain without the adverse risks associated with conventional analgesia.

In 2003, the World Health Organization approved acupuncture for postoperative pain control of low back pain [19]. The results of the present study on the use of acupuncture for pain control after surgery for degenerative spine disease are in many ways analogous to those of previous studies of the use of acupuncture to treat low back pain. After a review of the literature, Liu et al. [20] concluded that acupuncture, either used alone or as an adjunct to conventional therapy, provides short-term improvements in chronic low back pain, but studies with improved internal and external validity are still needed. Yuan et al. [21] conducted a systematic review of 23 trials with 6,359 patients and found moderate evidence that acupuncture is more effective than no treatment, and strong evidence of no significant differences between acupuncture and sham acupuncture for short-term pain relief. Furlan et al. [22] reviewed studies of complementary and alternative therapies for the treatment of neck and low back pain and reported that both complementary and alternative therapies were effective for immediate or short-term pain control, but that more rigorous studies of these methods are still needed.

The mechanism by which acupuncture can reduce pain is not fully understood. Acupuncture can relieve muscle pain through increasing the levels of endorphins, which are natural pain killers and stress fighters [23]. Another theory is that acupuncture stimulates the vagus nerve and decreases the inflammatory response to relieve chronic pain [23].

As results of the present study have shown, PCA is an effective method for pain control. A systematic review of randomized trials found that PCA provided better pain control than did traditional analgesic drugs [24]. Although PCA can maintain steady pain control, many patients develop nausea or vomiting while using it [25]. In the present study, the slightly higher pain scores in patients receiving PCA than in the other two groups was likely due to a “rebound” in pain after cessation of PCA after the third postoperative day, as patients’ VAS scores declined to those of patients in the other two groups thereafter.

This study has several limitations. First, it is a retrospective study with the inherent potential biases of that design. Second, it is a single-center study and thus the results may not be generalizable to other centers, populations, or acupuncture practitioners. Third, the use of NSAIDs and meperidine by some AC patients makes assessment of the possible benefit of acupuncture alone difficult in those patients. Fourth, although patients were able to choose their preferred pain control methods, patients still needed to pay an extra amount of approximately 300 US dollars for the PCA in compliance with the health insurance regulations in Taiwan, which may lead to selection bias.

## Conclusions

The results of this study show that acupuncture is as effective as PCA and routine analgesics for adjuvant pain control after open surgery for degenerative lumbar spine disease. Its efficacy compared with that of traditional analgesia and patient-controlled analgesia is not established because of methodologic problems with this study. Large, multi-center, rigorously controlled trials are needed to conclusively determine if acupuncture has a role in relieving pain in this patient population.

## Data Availability

The data used to support the findings of this study are included within the article.

## Abbreviations

AC: Acupuncture
NSAIDs: Non-steroid anti-inflammatory drugs
PCA: Patient-controlled analgesic
PreAC: Preoperative acupuncture
VAS: Visual analog scale
